# Parity is associated with cognitive function and brain age in both females and males

**DOI:** 10.1038/s41598-020-63014-7

**Published:** 2020-04-08

**Authors:** Kaida Ning, Lu Zhao, Meredith Franklin, Will Matloff, Ishaan Batta, Nibal Arzouni, Fengzhu Sun, Arthur W. Toga

**Affiliations:** 1 USC Stevens Neuroimaging and Informatics Institute, Keck School of Medicine of University of Southern California, Los Angeles, California 90033 USA; 2 Molecular and Computational Biology Program, University of Southern California, Los Angeles, CA 90089 USA; 3 Department of Preventive Medicine, University of Southern California, Los Angeles, CA 90032 United States; 4 Neuroscience Graduate Program, University of Southern California, Los Angeles, CA 90089 USA

**Keywords:** Cognitive ageing, Computational neuroscience

## Abstract

Previous studies of the association between parity and long-term cognitive changes have primarily focused on women and have shown conflicting results. We investigated this association by analyzing data collected on 303,196 subjects from the UK Biobank. We found that in both females and males, having offspring was associated with a faster response time and fewer mistakes made in the visual memory task. Subjects with two or three children had the largest differences relative to those who were childless, with greater effects observed in men. We further analyzed the association between parity and relative brain age (n = 13,584), a brain image-based biomarker indicating how old one’s brain structure appears relative to peers. We found that in both sexes, subjects with two or three offspring had significantly reduced brain age compared to those without offspring, corroborating our cognitive function results. Our findings suggest that lifestyle factors accompanying having offspring, rather than the physical process of pregnancy experienced only by females, contribute to these associations and underscore the importance of studying such factors, particularly in the context of sex.

## Introduction

Pregnancy involves dramatic hormonal and physiological changes. In part due to these large changes, the effect of pregnancy on cognitive and cardiovascular health has been studied^1,2^. Declined cognitive function was observed during pregnancy^1,2^. Researchers have also hypothesized that hormonal changes, which occur both during and after pregnancy, drive the association between parity (number of offspring) and cognitive function in later life. Multiple studies have investigated this association in females, though different conclusions have been found. Some studies found that parity was associated with better episodic memory and had a protective effect against Alzheimer’s disease (AD)^3,4^. Contrarily, parity has been associated with poor word recall score, Mini Mental State Exam score, and AD neural pathology^5,6^. A recent study of approximately 10,000 male and female subjects found an association between the number of offspring and cognitive function in later life in both sexes, including memory and executive function, and suggested that socioeconomic status largely accounted for the association^7^.

In addition to the association between cognitive function and parity, the association between brain structural change and parity has been studied previously. Hoekzema *et al*. reported that the volume of certain gray matter regions was reduced during pregnancy and the reductions did not recover for at least 2 years post partum^2^, while others reported that the gray matter restoration process was evident within the first few months postpartum^8,9^. Most studies on the association between brain structure and parity had a relatively small sample size (n < 100) and less than three years of postpartum follow-up^2,8,9^. To date, it is still unclear if there are any long-term effects of parity on brain structure in the mid-to-old age population. We therefore sought to investigate this question by studying the association of parity with a brain imaging-derived marker of aging. Recently, brain age, a metric derived from imaging data using machine learning techniques, has proven to be a promising biomarker for aging^10^. Advanced brain age is associated with AD, objective cognitive impairment, and schizophrenia, among other conditions^11–15^. We hypothesized that if there was a significant association between parity and wellbeing of the brain, there may also be an observable association between parity and brain age.

Further, having offspring leads to significant life changes in both females and males, all of which may impact the brain. For example, among low-parity men and women, more frequent use of alcohol and tobacco was observed^16^. Children might serve as a ‘bridge’ connecting parents to more social and community activities^17^. Adult children can provide parents with emotional and social support, as well as instrumental support such as s shopping and house work^18,19^. Modig *et al*. reported that having offspring was associated with lower mortality risk in both sexes. Interestingly, the differences in death risks between subjects with and without offspring were slightly larger for men than for women^20^. Therefore, we hypothesized that lifestyle and environmental factors accompanying having offspring, other than pregnancy history, might also play a role in the association between parity and wellbeing of the brain. In that case, an association between parity and wellbeing of the brain would be observed in both men and women. For the purposes of our study, we extended the definition of parity to be the number of offspring for both men and women.

In this study, we investigated the association between parity and cognitive function in both sexes using UK Biobank data, where data on visual memory, response time, demographic, and lifestyle information were collected on over 300,000 subjects of European ancestry. Further, we derived relative brain age (RBA) metric, a biomarker indicating the aging level of a person’s brain relative to peers, and studied how parity is associated with RBA using brain imaging data of over 13,000 subjects.

## Materials and methods

### Overview of study population

Our study population was obtained from the UK Biobank^21^ project, a large prospective cohort of approximately 500,000 participants who have provided demographic information as well as blood, urine and saliva samples. All participants provided informed consent; the present analyses were conducted under data application number 25641.

### Cognitive function data

Two important cognitive function scores, response time and visual memory, were available in more than 90% of UK Biobank subjects. Among subjects who completed both cognitive function tasks, we further limited the study sample to 303,196 subjects who were of European ancestry, didn’t have brain or nervous system related illness. Supplementary Table 1 lists diseases based on which subjects were excluded from our analyses.

Both cognitive function scores were test based, with response time representing the mean time for a subject to press a snap-button when two cards displayed on the computer screen matched. The unit of response time is millisecond. The visual memory test required subjects to memorize the position of matching pairs of cards shown on computer screen. Each subject was then instructed to select the pairs that were matching from memory after the symbols were subsequently hidden. Similar to previous research^22^, the recorded number of incorrect matches produces an overall score whereby lower scores indicate better visual memory with fewer errors in accomplishing the task. For our analysis the memory score was log-transformed after first adding the number 1 to it. Therefore, the unit of visual memory score used for analyses is log(number of mistakes made in memorizing matching cards). More details of the collection of cognitive function data can be found on the UK Biobank website (http://www.ukbiobank.ac.uk/).

### Obtaining relative brain age (RBA) based on magnetic resonance imaging (MRI) data

Quality controlled brain structural MRI data were available for 13,584 subjects. Brain morphometric measurements were obtained by processing MRI data with FreeSurfer 6.0^23^. More details including imaging hardware, acquisition protocols, and quality control are described elsewhere^23,24^.

We used a two-step regression approach to obtain relative brain age (RBA), a metric indicating how old a person’s brain structure appears relative to their peers, based on imaging data. First, we built a LASSO regression model for predicting brain age based on brain morphometric measurements. In this model, chronological age was response variable, brain morphometric measurements including volume of cortical, subcortical and white matter regions, thickness and surface area of cortical regions, ventricle size, intracranial volume, etc. were used as predictors. Supplementary Table 2 lists these morphometric measurements. Second, after obtaining predicted brain age (PBA) we regressed out age to form the RBA metric, which is orthogonal to age. To be specific, we observed that due to regression dilution^25^, the difference between PBA and CA (i.e., PBA - CA) was negatively associated with CA (see Supplementary Fig. 1). Therefore, after obtaining PBA for each subject, we further calculated RBA. RBA is defined as the difference between PBA and expected PBA given a subject’s chronological age (i.e., RBA = PBA- Expected(PBA|CA)). Here, Expected(PBA|CA)), or EPBA, was obtained through building a regression model where CA was the predictor and PBA was the response variable. In this way, RBA is independent of CA. At each age range, there were roughly half of the subjects with positive RBA and half of the subjects with negative RBA (see Supplementary Fig. 1). Since only linear operations were used to derive RBA based on PBA and CA, the unit of RBA is year. More details describing the process for calculating RBA based on brain MRI data have been described previously^24^.

### Demographic information

Demographic information, including parity (i.e., number of live births for women, and number of children fathered for men), age, education, body mass index (BMI), average total household income, past tobacco smoking frequency, alcohol intake frequency, sleep duration, living alone or with others, diabetes, and hypertension disease status, was obtained for all subjects. We do not have information regarding if both parents of the same children are recruited into the study. Parity was further categorized into 5 groups: no offspring, 1 offspring, 2 offspring, 3 offspring, and >=4 offspring. We treated number of offspring as a categorical variable rather than continuous for two reasons: first, the >=4 category contained subjects with 4, 5, or more offspring did not have a linear relationship with the other categories; second, we hypothesized that relationship of number of offspring with cognitive function and relative brain age might not be linear.

### Study the association between number of offspring and cognitive function

Linear regression with multivariable adjustment was used to study the association between cognitive function (response time and visual memory) and number of offspring separately for females and males. For both cognitive function outcomes we adjusted for age, education, body mass index (BMI), average total household income, past tobacco smoking frequency, alcohol intake frequency, sleep duration, living alone or with others, diabetes, and hypertension disease. Pairwise comparisons of number of offspring and each outcome were conducted using the Scheffe test. We conducted analyses for females and males separately first. We then combined data of both sexes, used an interaction term between sex and number of offspring to test whether the overall associations were significantly different for males and females.

### Study the association between number of offspring and RBA

**Figure 1:**
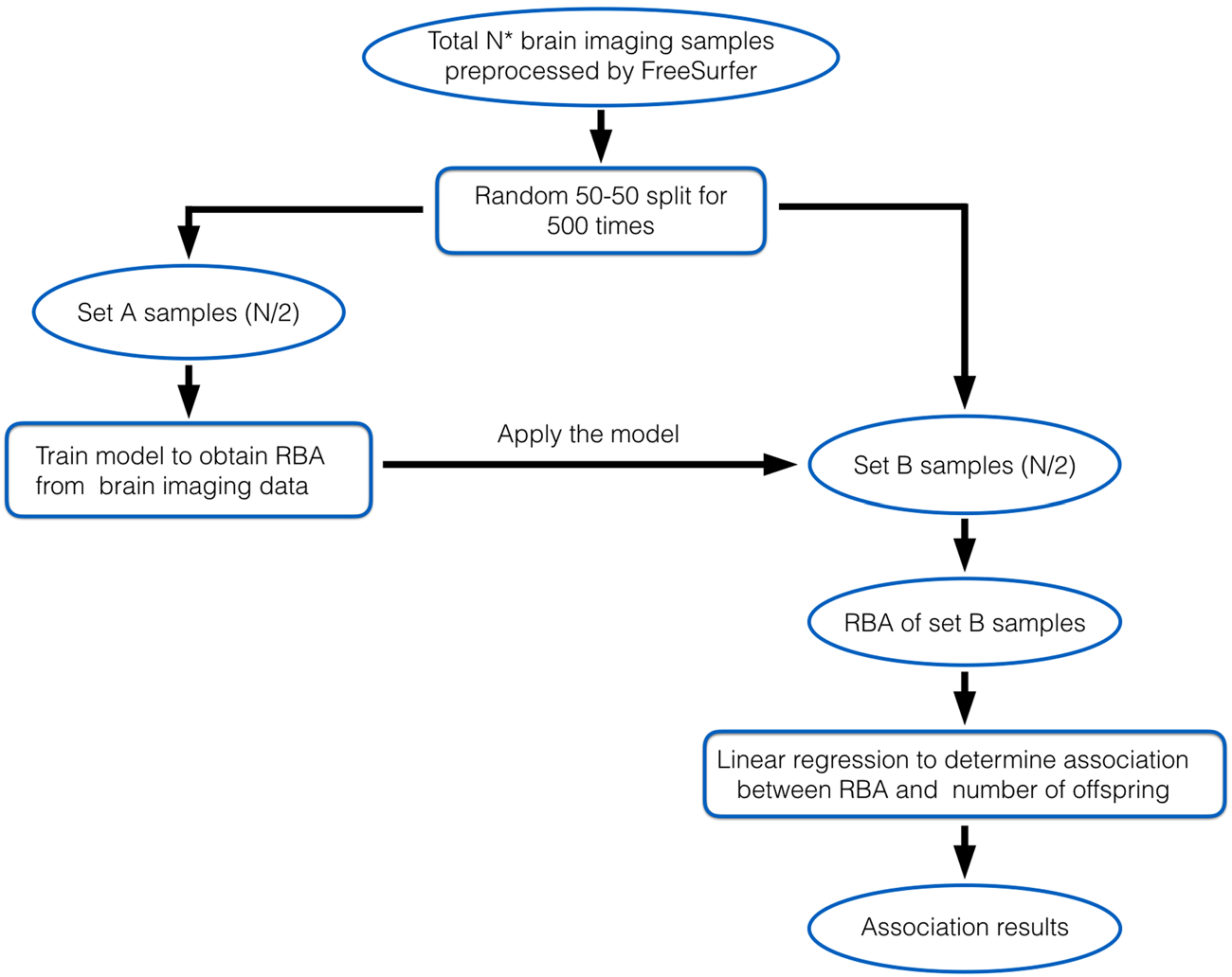
Procedure for studying the association between number of offspring and relative brain age using samplings. N = 6,822 for female subjects; N = 6,762 for male subjects.

We investigated the association between the number of offspring and RBA by repeating the following three-step procedure 500 times: First, we randomly split the samples into sets A and B, each having equal size. Second, using set A, we trained a model to obtain RBA based on MRI data and applied it to obtain RBA for set B. Third, using set B, we examined the association between number of offspring and RBA adjusting for the aforementioned covariates except for age, since RBA is orthogonal to age. The procedure was repeated 500 times so that distribution of the parameter of interest in all the rounds gave information on how sensitive the result is to the random splits used. The analyses procedure is visualized in a flowchart in Fig. 1. Similar to the analyses on the association between cognitive function and number of offspring, we analyzed the association in females and males separately and then combined the data of both sexes to look for interaction between sex and number of offspring in association with RBA.

Statistical significance was set at α = 0.05 and all regression analyses were conducted using the R language^26^.

## Results

### Descriptive results

Cognitive function data were available for 160,077 women and 143,119 men. Among female subjects, 19% were childless, 13% had one child, 46% had two children, 17% three children, and 5% four or more children. Among male subjects, 20% were childless, 13% had one child, 44% had two children, 17% three children, and 6% four or more children. Table 1 provides summary statistics all covariates considered in the analyses grouped by sex. Brain imaging data were obtained for 6,822 women and 6,762 men. Descriptive results for subjects with brain imaging data are shown in Supplementary Table 3.

**Table 1:** Demographic information of subjects included in the analyses for the association between parity and cognitive function.

	Female (n = 160,077)	Male (n = 143,119)
**Number of offspring, n (%)**		
0	29931 (18.7%)	28808 (20.1%)
1	20621 (12.9%)	18059 (12.6%)
2	72997 (45.6%)	63598 (44.4%)
3	27989 (17.5%)	24210 (16.9%)
>=4	8539 (5.3%)	8444 (5.9%)
Age, mean (SD)	56.7 (7.9)	57.5 (8.1)
**Education, n (%)**		
College or university degree	53736 (33.6%)	51636 (36.1%)
Other degree	106341 (66.4%)	91483 (63.9%)
**BMI, n (%)**		
Normal	64032 (40%)	35033 (24.5%)
Obese	36119 (22.6%)	35719 (25%)
Overweight	58910 (36.8%)	72143 (50.4%)
Underweight	1016 (0.6%)	224 (0.2%)
**Household income, n (%)**		
Less than 18,000	36161 (22.6%)	24930 (17.4%)
18,000 to 30,999	42706 (26.7%)	35129 (24.5%)
31,000 to 51,999	42046 (26.3%)	40240 (28.1%)
52,000 to 100,000	31273 (19.5%)	33775 (23.6%)
Greater than 100,000	7891 (4.9%)	9045 (6.3%)
**Past tobacco smoking, n (%)**		
Abstained from smoking	75167 (47%)	54420 (38%)
Just tried once or twice	26613 (16.6%)	23376 (16.3%)
Occasionally	22917 (14.3%)	20014 (14%)
On most or all days	35380 (22.1%)	45309 (31.7%)
**Alcohol intake, n (%)**		
Abstained from drinking	11034 (6.9%)	6084 (4.3%)
Special occasions only	20537 (12.8%)	8224 (5.7%)
1~3 times a month	21052 (13.2%)	12147 (8.5%)
1~2 times a week	43265 (27%)	37618 (26.3%)
3~4 times a week	36805 (23%)	41055 (28.7%)
Daily or almost daily	27384 (17.1%)	37991 (26.5%)
**Sleep duration, n (%)**		
Normal	122086 (76.3%)	108010 (75.5%)
Short	35724 (22.3%)	33248 (23.2%)
Long	2267 (1.4%)	1861 (1.3%)
**Living with others, n (%)**		
No	31468 (19.7%)	21886 (15.3%)
Yes	128609 (80.3%)	121233 (84.7%)
**Diabetes, n (%)**		
No	154801 (96.7%)	133868 (93.5%)
Yes	5276 (3.3%)	9251 (6.5%)
**Hypertension, n (%)**		
No	123179 (76.9%)	99636 (69.6%)
Yes	36898 (23.1%)	43483 (30.4%)

### Number of offspring and cognitive function

**Figure 2:**
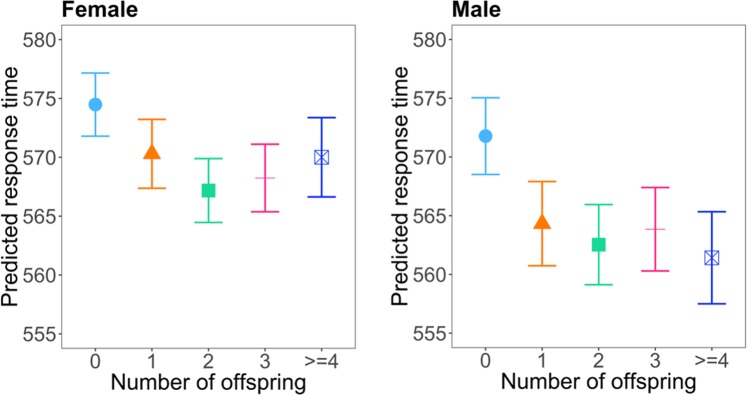
Number of offspring versus response time predicted by model with multivariable adjustment in female (left) and male (right) subjects. The unit of response time is millisecond.

**Figure 3:**
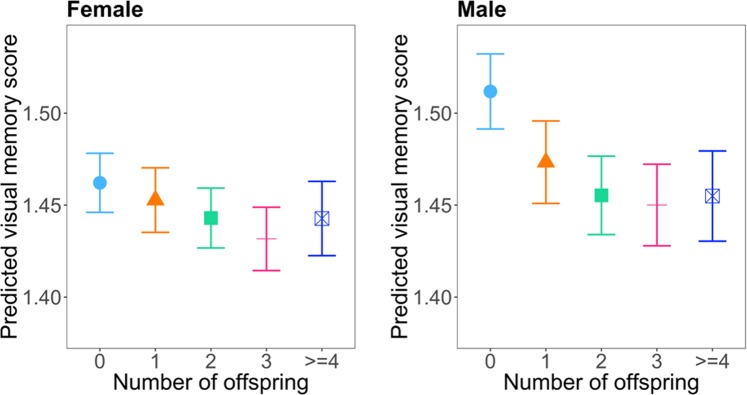
Number of offspring versus visual memory score in female (left) and male (right) subjects. The unit of visual memory is log(number of mistakes made in memorizing matching cards).

In female subjects, the number of offspring was statistically significantly associated with both response time and visual memory according to regression models that adjusted for covariates as described in the methods section (ANOVA F-test p-values <0.001). Compared with subjects who were childless, those with any number of offspring had shorter response time and made fewer mistakes in visual memory task. A non-linear relationship was observed between number of offspring and response time and between number of offspring and visual memory, confirmed with statistically significant quadratic trend tests (p-value = 0.002 for response time, and p-value <0.001 for visual memory). Figures 2 and 3 illustrate these trends, and parameter estimates for number of offspring in the two models are listed in Tables 2 and 3, respectively. To further assess the magnitude of the association of parity with cognitive function we compared the coefficient of parity and the coefficient of age in the regression models. The coefficient column in Table 2 shows that female subjects with one offspring had a response time that was 4.2 milliseconds faster than childless female subjects. As a comparison, according to the regression model, each year of increased age was associated with 4.2 milliseconds of longer response time in females. Therefore, compared to females with no offspring, females with one offspring had response time reduction that was similar to that of a one-year reduction of age. We had similar observation for the association between parity and visual memory score. Table 3 shows that females with one offspring had decreased (better) visual memory score of 0.01 compared to those with no offspring. Comparatively, each year of increased age was associated with an increased (worse) visual memory score of 0.01 in females. Therefore, compared to females with no offspring, females with one offspring had a visual memory improvement that was similar to that of a one-year reduction of age. Pairwise comparisons among parity groups showed that subjects with 2 or 3 children and those who were childless had the largest differences in both response time and visual memory, although for visual memory score among females the difference did not reach statistical significance when adjusted for multiple testing. Pairwise differences among those with offspring were small and non-significant (Supplementary Table 4).

**Table 2:** Coefficient estimations of number of offspring in association with response time in regression model with multivariable adjustment.

Female		Male	
Childless (baseline)	Coefficient (95% CI)	Childless (baseline)	Coefficient (95% CI)
1 offspring	−4.18 (−6.05,−2.31)**	1 offspring	−7.45 (−9.4,−5.50)**
2 offspring	−7.30 (−8.77,−5.83)**	2 offspring	−9.24 (−10.76,−7.71)**
3 offspring	−6.24 (−8.00,−4.47)**	3 offspring	−7.93 (−9.76,−6.09)**
>=4 offspring	−4.47 (−7.02,−1.93)**	>=4 offspring	−10.36 (−12.9,−7.81)**

**p-value<0.001.

The unit of response time is millisecond.

**Table 3:** Coefficient estimations of number of offspring in association with visual memory score.

Female		Male	
Childless (baseline)	Coefficient (95% CI)	Childless (baseline)	Coefficmorent (95% CI)
1 offspring	−0.01 (−0.02,0.00)	1 offspring	−0.04 (−0.05,−0.03)**
2 offspring	−0.02 (−0.03,−0.01)**	2 offspring	−0.06 (−0.07,−0.05)**
3 offspring	−0.03 (−0.04,−0.02)**	3 offspring	−0.06 (−0.07,−0.05)**
>=4 offspring	−0.02 (−0.03,0.00)*	>=4 offspring	−0.06 (−0.07,−0.04)**

*P-value<0.05.

**P-value<0.001.

The unit of visual memory is log(number of mistakes made in memorizing matching cards).

In male subjects, the number of offspring was also significantly associated with both response time and visual memory (ANOVA F-test p-values <0.001). Compared with subjects who were childless, those with offspring had shorter response time and made fewer mistakes in visual memory task. Similar to females, a quadratic trend existed for the associations with both outcomes (p-value < 0.001) as shown in Figs. 2 and 3 and parameter estimates for number of offspring are listed in Tables 2 and 3. Comparing coefficients of offspring and age in the regression model for male subjects, we found that compared to males with no offspring, subjects with one offspring had response time reduction that was similar to that of a two-year reduction of age, and had a visual memory improvement similar to a four-year reduction of age. Pairwise comparisons among groups with different number of offspring also showed that the strongest difference was between those with 2 or 3 children and those who were childless, while the difference between those with offspring was small for both response time and visual memory (Supplementary Table 4).

Regression models with integrated data from both sexes indicated significant interaction between number of offspring and sex on response time and visual memory, where protective effects of having offspring on cognitive function appeared to be larger in male subjects than female subjects (p-value of interaction <0.001 for both cognitive functions).

### Number of offspring and relative brain age (RBA)

**Figure 4:**
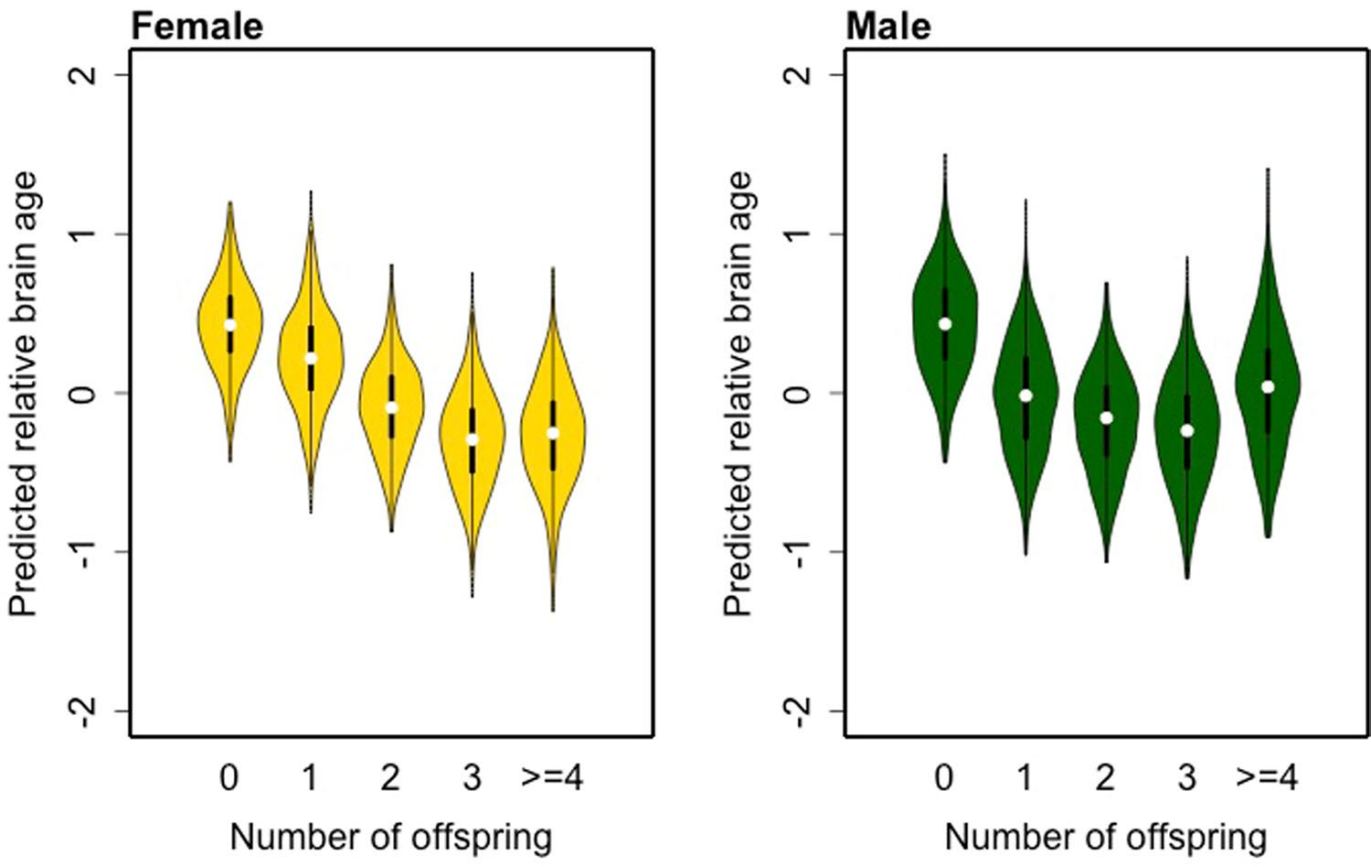
Distribution of relative brain age (RBA) predicted by model with multivariable adjustment over 500 samplings in female (left) and male (right) subjects. White points represent the median values of the estimate in each group with different number of offspring. The unit of RBA is year.

The number of offspring was significantly associated with RBA in both sexes. In 500 random samplings, median ANOVA p-value for the association between number of offspring and RBA was <0.001 for both female and male subjects. Among females, compared with those who were childless, subjects with two offspring were estimated to have a brain age that was 0.5 years younger, and subjects with three offspring were estimated to have a brain age that was 0.7 years younger. Among males, subjects with two offspring were estimated to have a brain age that was 0.6 years younger, and subjects with three offspring were estimated to have a brain age that was 0.7 years younger. In female subjects, a significant linear trend (p < 0.001) of the association was observed, while in male subjects a quadratic trend (p < 0.001) was observed (Fig. 4). Table 4 shows the median parameter estimates for the number of offspring across the 500 samplings. No significant interaction was observed between number of offspring and sex on RBA.

**Table 4:** Median of coefficient estimations for number of offspring in association with relative brain age (RBA) in regression model with multivariable adjustment in 500 samplings.

Female		Male	
Childless (baseline)	Coefficient (95% CI)	Childless (baseline)	Coefficient (95% CI)
1 offspring	−0.21 (−0.66,0.24)	1 offspring	−0.46 (−0.93,0.01)
2 offspring	−0.52 (−0.87,−0.17)*	2 offspring	−0.62 (−0.99,−0.25)*
3 offspring	−0.72 (−1.15,−0.29)*	3 offspring	−0.68 (−1.13,−0.23)*
>=4 offspring	−0.69 (−1.36,−0.02)*	>=4 offspring	−0.41 (−1.06,0.24)

*p-value<0.05.

95% CI is inferred from the median of the standard error among 500 samplings.

The unit of RBA is year.

## Discussion

We studied the association of number of offspring with response time, visual memory, and brain imaging derived RBA in the UK Biobank cohort.

We observed that in both males and females, having offspring is associated with better visual memory and faster response time after adjusting for age, education, BMI, income, smoking, and other factors. It is possible that having a child drives these associations. One possibility is that having offspring is associated with significant life changes, which may improve brain health directly or indirectly. First, having offspring is associated with healthier lifestyle, such as less frequent use of alcohol and tobacco and more regular meal times^16,27^. Second, children might serve as a ‘bridge’ connecting parents to more social and community activities^17^, which improves cognitive function^28,29^. Third, adult children can provide parents with emotional and social support, as well as instrumental support such as shopping and house work^18,19^. However, we also acknowledge that it is possible that the aforementioned beneficial effect of having offspring does not monotonically increase as the number of children increases. In addition, child rearing is also associated with increased financial and physical stress^30,31^. As shown in previous studies, parity is associated with increased cardiovascular disease risk and increased BMI in both sexes^32,33^. This could possibly explain our observation of a “U-shape” association; cognitive function did not monotonically improve with increasing number of offspring.

Further, the association between cognitive function and parity was significantly stronger in male subjects than in female subjects. Since males do not experience the physical process of pregnancy, our observation further suggests that lifestyle factors accompanying having offspring may play an important role in the association between parity and cognitive function. Our finding is corroborated by previous studies. Zhang *et al*. that showed that single men who were childless had significantly higher rates of loneliness and depression compared with women in comparable circumstances^34^. Modig *et al*. reported that having offspring was associated with lower mortality risk in both sexes. Interestingly, the differences in death risks between subjects with and without offspring were slightly larger for men than for women^20^.

Most previous studies on the long-term association between parity and wellbeing of the brain only evaluated cognitive function^1,3,4,6^. Our study contributes new information because we further looked into the association between parity and RBA, a biomarker of structural aging of the brain, and observed findings that corroborated the association between parity and cognitive function. In both sexes, subjects with any number of offspring had younger appearing brain than subjects with no offspring. In male subjects, the association between parity and RBA followed a “U-shape” pattern, where subjects with 2 or 3 offspring had younger appearing brain compared to subjects with 0, 1, or >=4 offspring. That was similar to the association observed between parity and cognitive function. In comparison, a linear relationship was observed between number of offspring and RBA in females. This linear association may be explained by the hormonal fluctuation specifically linked to women’s pregnancy history and remains to be further investigated.

Relative strengths of the study are its large sample size, inclusion of both male and female subjects, and observation in the association between RBA and parity that further supported the association between cognitive function and parity. Our study also has a few limitations. First, while we adjusted for a number of socioeconomic, lifestyle, and health covariates in our model, we were not able to account for the possibility that they may be time varying. Details of these covariates in early life could be useful for understanding other underlying issues related to cognitive function and structural aging of the brain. Second, the study is an observational study, so it is impossible to conclude that having offspring is leading to improved brain health. It could also be possible that those who have poor underlying health have fewer opportunities to have offspring. Third, since only a small proportion of subjects had 5 or more children, we categorized number of off-spring into 0, 1, 2, 3, and >=4 as in previous studies using this variable^7,32^, and did not study the difference among those who have 4, 5, or more offspring. Fourth, brain health is only a small part of overall health condition of the body. Although we found that having offspring is associated with better visual memory, faster response time, and a younger looking brain, we may not conclude that having offspring is associated with improved wellness of the whole body.

In conclusion, we observed robust evidence that parity is associated with visual memory, response time, as well as RBA in both sexes. Our observation suggests that lifestyle factors associated with having offspring, likely shared by both sexes, contribute to these associations. At the same time, we observed different detailed association patterns within women and men, which suggest the importance of studying the association between parity and wellbeing of the brain in the context of sex.

## Supplementary information

Supplementary Information.

Supplementary Table S1

Supplementary Table S2

SupplementaryTable S3

Supplementary Table S4

## Data Availability

UK Biobank data can be accessed through a procedure described at http://www.ukbiobank.ac.uk/using-the-resource/.

## References

[1] Davies SJ, Lum JA, Skouteris H, Byrne LK, Hayden MJ (2018). Cognitive impairment during pregnancy: a meta-analysis. Med J Aust.

[2] Hoekzema E (2017). Pregnancy leads to long-lasting changes in human brain structure. Nat Neurosci.

[3] Henderson VW, Guthrie JR, Dudley EC, Burger HG, Dennerstein L (2003). Estrogen exposures and memory at midlife: a population-based study of women. Neurology.

[4] Fox M, Berzuini C, Knapp LA (2013). Cumulative estrogen exposure, number of menstrual cycles, and Alzheimer’s risk in a cohort of British women. Psychoneuroendocrinology.

[5] Beeri MS (2009). Number of children is associated with neuropathology of Alzheimer’s disease in women. Neurobiol Aging.

[6] Heys M (2011). Life long endogenous estrogen exposure and later adulthood cognitive function in a population of naturally postmenopausal women from Southern China: the Guangzhou Biobank Cohort Study. Psychoneuroendocrinology.

[7] Read SL, Grundy EMD (2017). Fertility History and Cognition in Later Life. J Gerontol B Psychol Sci Soc Sci.

[8] Luders E (2018). Potential Brain Age Reversal after Pregnancy: Younger Brains at 4-6Weeks Postpartum. Neuroscience.

[9] Oatridge A (2002). Change in brain size during and after pregnancy: study in healthy women and women with preeclampsia. AJNR Am J Neuroradiol.

[10] Cole JH (2017). Neuroimaging-derived brain-age: an ageing biomarker?. Aging (Albany NY).

[11] Franke K, Ziegler G, Kloppel S, Gaser C, Alzheimer’s Disease Neuroimaging I (2010). Estimating the age of healthy subjects from T1-weighted MRI scans using kernel methods: exploring the influence of various parameters. Neuroimage.

[12] Franke K, Gaser C, Manor B, Novak V (2013). Advanced BrainAGE in older adults with type 2 diabetes mellitus. Front Aging Neurosci.

[13] Nenadic I, Dietzek M, Langbein K, Sauer H, Gaser C (2017). BrainAGE score indicates accelerated brain aging in schizophrenia, but not bipolar disorder. Psychiatry Res.

[14] Cole JH, Franke K (2017). Predicting Age Using Neuroimaging: Innovative Brain Ageing Biomarkers. Trends Neurosci.

[15] Liem F (2017). Predicting brain-age from multimodal imaging data captures cognitive impairment. Neuroimage.

[16] Kravdal O (1995). Is the relationship between childbearing and cancer incidence due to biology or lifestyle? Examples of the importance of using data on men. Int J Epidemiol.

[17] Furstenberg, F. F. Banking on families: how families generate and distribute social capital *Journal of marriage and family* **67** (2005).

[18] Kramarow, E. A., Lentzner, H. R., Rooks R. N., Weeks, J. D. & Saydah, S. H. Health, United States. (1999).

[19] Ross CE, Mirowsky J (2002). Family relationships, social support and subjective life expectancy. J Health Soc Behav.

[20] Modig K, Talback M, Torssander J, Ahlbom A (2017). Payback time? Influence of having children on mortality in old age. J Epidemiol Community Health.

[21] Allen NE, Sudlow C, Peakman T, Collins R, Biobank UK (2014). UK biobank data: come and get it. Sci Transl Med.

[22] Davies G (2016). Genome-wide association study of cognitive functions and educational attainment in UK Biobank (N = 112 151). Mol Psychiatry.

[23] Fischl B (2012). FreeSurfer. Neuroimage.

[24] Ning, K., Zhao, L., Matloff, W., Sun, F. & Toga, A. W. Association of brainage with smoking, alcohol consumption, and genetic variants. *bioRxiv* (2018). 10.1038/s41598-019-56089-4 PMC6992742 32001736

[25] Hutcheon JA, Chiolero A, Hanley JA (2010). Random measurement error and regression dilution bias. BMJ.

[26] R Core Team. R: A language and environment for statistical computing. *R Foundation for Statistical Computing* (2012).

[27] Kendig, H., Dykstra, P. A., van Gaalen, R.I . & Melkas, T. Health of aging parents and childless individuals. *Journal of Family Issues* **28** (2007).

[28] Wang HX, Karp A, Winblad B, Fratiglioni L (2002). Late-life engagement in social and leisure activities is associated with a decreased risk of dementia: a longitudinal study from the Kungsholmen project. Am J Epidemiol.

[29] Yen YC, Yang MJ, Shih CH, Lung FW (2004). Cognitive impairment and associated risk factors among aged community members. Int J Geriatr Psychiatry.

[30] Blanchflower, D. G. & Clark, A. E. Children, Unhappiness and Family Finances: Evidence from One Million Europeans. *National Bureau of Economic Research* **Working****Paper 25597**(2019).

[31] Richter, D., Kramer, M. D., Tang, N. K. Y., Montgomery-Downs, H. E. & Lemola, S. Long-term effects of pregnancy and childbirth on sleep satisfaction and duration of first-time and experienced mothers and fathers. *Sleep* **42** (2019). 10.1093/sleep/zsz015 30649536

[32] Magnus MC (2017). Number of Offspring and Cardiovascular Disease Risk in Men and Women: The Role of Shared Lifestyle Characteristics. Epidemiology.

[33] Peters SA, Huxley RR, Woodward M (2016). Women’s reproductive health factors and body adiposity: findings from the UK Biobank. Int J Obes (Lond).

[34] Zhang Z, Hayward MD (2001). Childlessness and the psychological well-being of older persons. J Gerontol B Psychol Sci Soc Sci.

