# Supplementary material for: Parity is associated with cognitive function and brain age in both females and males
Source: Sci Rep. 2020 Apr 8;10:6100. doi: 10.1038/s41598-020-63014-7 (PMC7142076; doi:10.1038/s41598-020-63014-7)
Supplement: Supplementary file 1 — Supplementary Information. [file 41598_2020_63014_MOESM1_ESM.pdf]

Supplementary Information

Parity is associated with cognitive function and brain age in both females and males

Kaida Ning [a, b], Lu Zhao [a], Meredith Franklin [c], Will Matloff [a,d], Ishaan Batta [a], Nibal Arzouni [a, b], Fengzhu Sun [b], Arthur W. Toga [a, *]

[a] USC Stevens Neuroimaging and Informatics Institute, Keck School of Medicine of University of Southern California, Los Angeles, California 90033, USA

[b] Molecular and Computational Biology Program, University of Southern California, Los Angeles, CA 90089, USA

[c] Department of Preventive Medicine, University of Southern California, Los Angeles, CA 90032, United States

[d] Neuroscience Graduate Program, University of Southern California, Los Angeles, CA 90089, USA

Corresponding author: Arthur W. Toga
*Corresponding author at: USC Stevens Neuroimaging and Informatics Institute, Keck School of Medicine of University of Southern California, 2025 Zonal Ave., Los Angeles, California 90033, USA. Tel.: +1 323 442 7246
Fax: +1 323 442 0137
E-mail address: Toga@loni.usc.edu

Supplementary Figure

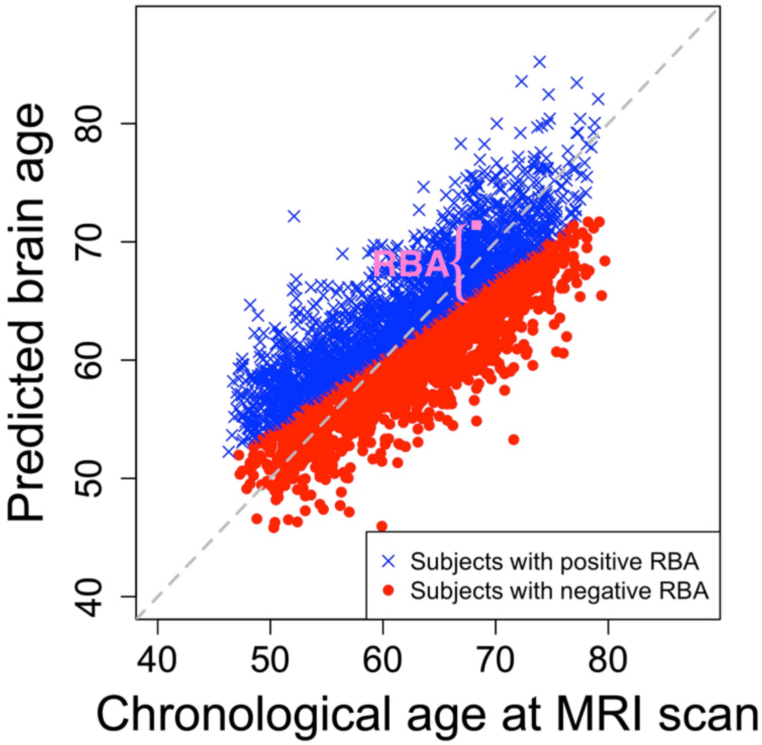

Supplementary Figure 1. Relationship between chronological age and the predicted brain age in the evaluation set in one sampling. Subjects with positive relative brain age (RBA) are labeled with blue X's; subjects with negative RBA are labeled with red dots. The RBA of a specific subject is illustrated in pink.

Supplementary Tables

Supplementary Table 1. List of diseases based on which subjects were excluded from the analyses.

Supplementary Table 2. List of brain morphometric measurements used for obtaining relative brain age.

Supplementary Table 3. Demographic information of subjects included in the analyses for the association between relative brain age and parity.

Supplementary Table 4. Pairwise differences among subjects with different number of offspring.
